# Emerging Burkholderia Musculoskeletal Infections With Delayed Diagnosis in Non-endemic Regions Affect Patient Morbidity: A Case Series of 10 Patients With a Review of the Literature

**DOI:** 10.7759/cureus.53096

**Published:** 2024-01-28

**Authors:** Sandeep Kumar, Vivek Bhambhu, Shataayu Gugale, Rohit Goyal, Anchin Kalia

**Affiliations:** 1 Orthopaedics, Mahatma Gandhi Medical College and Hospital, Jaipur, IND; 2 General Medicine, Mahatma Gandhi Medical College and Hospital, Jaipur, IND

**Keywords:** emerging infections, musculoskeletal infections, melioidosis, non-endemic regions, burkholderia species

## Abstract

Introduction: Burkholderia infection commonly presents as bacteraemic pulmonary disease; however, it is notorious for its wide variety of presentations in chronic cases, including musculoskeletal manifestations. It is common in patients living in endemic areas with comorbidities such as diabetes and who have chronic alcoholism. It was previously under-reported due to a low index of suspicion. Now, there is an increasing trend of diagnosis of these infections in non-endemic areas because of various factors, such as MALDI-TOF, molecular tests, and PCR.

Materials and methods: This is a single tertiary centre study of 10 patients, diagnosed with Burkholderia infection and treated at our institution between 2021 and 2023 and followed up for a minimum of six months. Information was collected from outpatient and inpatient records.

Results: In this study, the mean age of the patients was 45 years, with eight males and two females. Out of 10, seven patients had comorbidities. However, only one patient has a history of travelling to an endemic area. All our patients were treated operatively, and the course of intervention and the planning of the surgical procedure were decided according to clinico-radiological findings. Six out of 10 patients suffering from Burkholderia species infections have a history of prolonged ICU stay, four of them tested positive for Burkholderia pseudomallei and the remaining two tested positive for Burkholderia cepacia,* *with a mean average time of 24.6 days. Diabetes was the most common comorbidity in 70% of the patients. The knee was the most commonly affected joint, showing involvement in 60% of patients. We have done surgical intervention in all patients. In our study, we have given IV antibiotics for a minimum of six weeks to all patients, followed by oral antibiotic therapy for three to six months on the basis of regular follow-up of clinico-haematologic parameters.

Conclusion: Infections caused by Burkholderia species should be considered a potential causative agent of musculoskeletal infections in non-endemic areas without prior history of travelling to endemic areas. It may present with a chronic, mild course; a high index of suspicion is required, and it is important that due suspicion translates to prompt diagnosis and appropriate treatment to mitigate the course of the disease and associated morbidities in patients.

## Introduction

Burkholderia infection, while being endemic to northern Australia and South East Asia, is considered to be of emerging significance in non-endemic areas and in those returning or travelling from melioidosis-endemic regions [1]. Although it commonly presents as bacteraemic pulmonary disease, melioidosis is notorious for its wide variety of presentations, including musculoskeletal manifestations [2].

The Burkholderia genus, based on the description, included only seven organisms and currently includes a total of 56 species. These organisms’ existence is mainly based on the pH of the soil, and this constitutes their source of formation. They have an intrinsic acid tolerance and proliferate in acidic soils. It produces non-haemolytic greyish-white colonies on blood agar plates, and it is a non-fermenting, bipolar-staining bacillus [3].

Burkholderia can be divided into two main lineages that represent unique lines of descent [4]. One group of pathogens infects humans, animals, and plants, which includes Burkholderia pseudomallei, Burkholderiamallei, and Burkholderia glumae. B. pseudomallei primarily causes infections in animals and humans (melioidosis) [3]. Another clade comprised Burkholderia cepacia, initially known to cause rotting in onion bulbs, is a group of currently 20 closely related bacterial species in particular, named B. cepacia complex, which have emerged as opportunistic pathogens and are known to cause severe infections in immunocompromised patients [3].

Melioidosis can present with diverse clinical manifestations, including pneumonia, genitourinary infection, internal organ abscesses, septic arthritis, neurological melioidosis, and fulminant septicaemia without evident focus [5]. It is more common in patients with diseases such as diabetes, chronic alcohol intake, chronic liver disease, carcinomas, renal failure, blood disorders, and cardiac diseases. The most important predisposing risk factor is diabetes mellitus, multiplying the risk by 100 times [1].

This infection was previously under-reported due to low suspicion and the lack of diagnostic modalities [6]. Now, there is an increasing trend of diagnosis of these infections in non-endemic areas because of better diagnostic facilities, communication betterments due to technological advancements, and increased incidence of travel within the population [7].

## Materials and methods

This is a single tertiary centre study of 10 patients, diagnosed with musculoskeletal infection with Burkholderia species. Treatment was given at our institution between 2021 and 2023. The follow-up period of all the patients was for a minimum of six months. Statistical data were collected from inpatient records. Patient details, such as demographic data, history, clinical manifestations, comorbidities, and medical and surgical treatment were documented.

Pathological samples were collected from all the patients during surgical procedures. Adherence to best clinical methods was taken care of during sampling, which includes sample collection in proper and adequate amounts. Sterility was maintained throughout while in the transport chain. Appropriate media and antimicrobial disc were used for microbiological confirmation. Guidelines of the Clinical Laboratory Standards Institute (CLSI) were used in antimicrobial susceptibility testing [8]. Antimicrobial susceptibility testing was performed. B. pseudomallei is Gram-negative bacilli on Gram staining.

To analyse the results, categorical variables being represented as raw values/percentages and continuous variables represented as averages and ranges have been used. Consent taken from each subject regarding the data for this study would be used for publication, and approval was taken from the Institutional Research Ethics Committee of Mahatma Gandhi Medical College and Hospital, Jaipur, India (approval no. MGMC&H/IEC/JPR/2023/1037). No external source of funding was utilised for this study. In the current study, we aimed to describe the emergence of Burkholderia infections, including musculoskeletal manifestations in non-endemic regions with analysis of medical and surgical interventions.

## Results

In this series, the patients’ mean age was 45 years, the youngest being 20 years of age and the oldest being 57 years of age. Eighty percent of the patients were males, and the remaining were females. All 10 patients had musculoskeletal symptoms. However, four of them also had systemic manifestations. Six patients had a history of prolonged ICU admission, with a mean average time of 24.6 days, for systemic illness; out of these six, two patients tested positive for B. cepacia complex and four for B. pseudomallei. Of the remaining four patients not admitted to the ICU, two tested positive for B. cepacia complex and two for B. pseudomallei. Seven patients out of the total 10 had occupations involving contact with soil, such as labourers and farmers, as mentioned in Table 1.

**Table 1:** Patient-specific data ICU - Intensive care unit; IV - Intravenous; Rt - Right; Lt - Left; B/L - Bilateral; DM - Diabetes mellitus; HTN - Hypertension; M - Male; F - Female

Serial Number	Age (Years)	Sex	Occupation	Features (Musculoskeletal/Systemic)	Comorbidities	Travel History to an Endemic Area	ICU Stay	Joint Involved	Treatment (Surgical)	Culture	Antibiotic Duration	Follow-up
1	46	M	Housework	Both	DM/HTN	No	30d	Rt ankle	Arthroscopic synovectomy	Burkholderia pseudomallei	IV(45 days)+Oral (100 days)	8 months
2	57	M	Business	Both	HTN/DM/Thyroid	No	25d	Rt shoulder/Rt ankle	Incision and drainage	Burkholderia pseudomallei	IV(42 days)+Oral(96 days)	6 months
3	35	M	Farmer	Musculoskeletal	Nil	No	-	Rt knee	Arthroscopic synovectomy	Burkholderia pseudomallei	IV(46 days)+Oral(120 days)	12 month
4	57	M	Policeman	Musculoskeletal	DM	No	-	Lt knee(Femur+Tibia bone infarcts)	Arthrotomy	Burkholderia cepacia complex	IV(42 days) + Oral (98 days)	12 month
5	20	M	Farmer	Musculoskeletal	Nil	No	26d	Lt shoulder+Lt knee	Arthroscopic synovectomy	Burkholderia cepacia	IV(42 days) + Oral (100 days)	7 months
6	52	F	Labourer	Both	HTN/DM	No	-	Rt shoulder	Incision and drainage	Burkholderia pseudomallei	IV(44 days)+Oral(110 days)	1.5 years
7	55	M	Farmer	Both	DM	No	-	Rt knee	Arthrotomy	Burkholderia cepacia complex	IV(43 days) + Oral (100 days)	2 years
8	43	F	Bank job	Musculoskeletal	Nil	Yes	25d	B/L knees	Arthroscopic synovectomy	Burkholderia pseudomallei	IV (42 days) + Oral (120 days)	1 year
9	56	M	Shopkeeper	Musculoskeletal	DM	No	15d	Lt shoulder	Arthrotomy	Burkholderia pseudomallei	IV (45 days)+Oral(100 days)	8 months
10	29	M	Farmer	Musculoskeletal	DM	No	27d	B/L knees	Arthroscopic synovectomy	Burkholderia cepacia	IV (43 days) + Oral (112 days)	8 months

On the evaluation of the patients, seven patients had a significant history of comorbidities. All seven patients had type 2 diabetes mellitus, two patients had hypertension, and one patient had hypothyroidism. Thus, overall, the most common comorbidity in our patients was diabetes mellitus (70%). Out of 10 patients, only one patient had a travel history to an endemic area (Australia). Five patients had multiple joint involvement, and one patient presented with primary osteomyelitis of the left distal femur and left proximal tibia. The most common joint is the knee (60%), followed by the shoulder and ankle, as mentioned in Table 1.

All our patients were treated operatively, and the course of intervention and the planning of the surgical procedure were decided according to clinico-radiological findings. Operative intervention was chosen when conservative treatment with regard to joint aspiration and administration of medications failed. In five of them, arthroscopic synovectomy was done, three were operated for incision and drainage, and in two patients arthrotomy was performed. Pus and tissue samples were collected and sent for microbiological evaluation. Antibiotics were given according to culture and sensitivity. Sensitivities were most common for cephalosporins (average zone diameter of 21 mm), beta-lactamases (average zone diameter of 23 mm), carbapenems (average zone diameter of 27 mm), fluoroquinolones (average zone diameter of 22 mm), and tetracyclines (average zone diameter of 18 mm). Intravenous antibiotics were given for a minimum of six weeks (average: 6.2 weeks), followed by oral antibiotics (average: 105.6 days). All patients were followed up at one week and then subsequently at every two-week intervals (average: 11.5 months), till they became free of clinical symptoms and inflammatory markers showed a decreasing trend, ultimately lying within normal ranges.

## Discussion

Melioidosis, caused by B. pseudomallei, is a facultative intracellular Gram-negative, saprophytic bacterium. It is an endemic disease in northern Australia and Southeast Asian countries, especially Thailand and Malaysia. It can virtually affect any organ in the body and can present with diverse clinical manifestations. Despite improvements in antimicrobial therapy, melioidosis is still associated with high morbidity and mortality [5].

The mean age of presentation in our study was 45 years, which is almost similar to other studies, such as that of Shetty et al., who stated that the median age at presentation was 48 [9].

The majority of the patients are males (80%), which is similar to a study conducted by Pandey et al., wherein they reported a series of four cases, in which three out of the four patients under consideration for the study were males [10]. Morse et al. reported that almost 55% of the patients under consideration in their study were males [11]. In our study also, the predisposition for male patients is noted, and this might be likely due to males being involved more in outdoor activities as compared to females. Seventy percent of our patients also have a history of contact with soil in their occupation, which is similar to the study conducted by Eberl et al. [3]. Direct contact with soil predisposes patients to a higher risk of contracting the disease [12].

In our study, we have noticed that six out of 10 patients suffering from Burkholderia species infections have a history of prolonged ICU stay, with a mean average time of 24.6 days. Raja et al. reported that an important mode of transmission of B. pseudomallei infections is the spread via bloodstream by percutaneous inoculation such as IV lines, central venous catheters, etc. [12]. Morse et al. reported that B. cepacia has emerged as an opportunistic nosocomial pathogen since the 1980s, particularly in patients with debilitating diseases and a long history of ICU stay [11]. Ingestion and inhalation are also suggested to be likely causes, particularly during damp weather conditions. In our study, no significant history relating to the transfer of the pathogen from soil could be isolated; however, most of the patients were either labourers or farmers from rural areas [12]. Pandey et al. said that it has got widespread occurrence and is found in the soil of almost all the states of India. However, it is reported more from southern states [10].

With regard to comorbidities, Shetty et al. reported that a total of 40 patients had one or more comorbidities. Diabetes was the most common among them, associated with multifocal involvement [9]. Perumal et al. stated that at least one comorbidity in 20 and multiple comorbidities were present in six patients. Diabetes was the most common comorbidity [13]. Our study has similar findings wherein 70% of patients had diabetes.

When considering systemic infections and manifestations, Perumal et al. reported that 37 (9.2%) had musculoskeletal involvement. Four patients (15%) had multisystem involvement in 37 musculoskeletal infective patients [13]. Morse et al., in their study from Northern Australia, stated that out of 41 patients with musculoskeletal manifestations, 14 patients had primary melioidosis [12]. In our study, we have systemic manifestations in four patients out of 10.

In our study, the knee was the most commonly affected joint, showing involvement in 60% of patients, which is very similar to the study conducted by Raja et al., where the knee was the most affected joint [12]. However, in their study, Kosuwon et al. found the shoulder to be the most commonly involved joint [14]. Perumal et al. stated that the lower limb was more involved than the upper limb musculoskeletal system, which is similar to our results. Multiple musculoskeletal foci of infection were also noted in a study conducted by Perumal et al. [13] and Punyagupta et al. [15]. Of our patients, 30% patients had multiple foci of infection, and the remaining presented with isolated joint involvement.

In terms of operative intervention for the disease, according to Perumal et al., all patients required operative intervention with medical treatment [13]. Morse et al. [11] did a study of 20 patients with musculoskeletal Burkholderia infections and found that adequate surgical drainage and debridement are vital, along with appropriate intravenous antibiotics. Subhadrabandhu et al. [16] published a series of 10 patients, and Pandey et al. [10] published a series of five patients, respectively, stating treatment strategies of antibiotic administration in addition to surgical debridement. Shetty et al. stated that 35 patients were operated on in the form of debridement. Twelve were minor procedures, out of a total of 63 admissions, and 16 were treated non-operatively. Patients with musculoskeletal manifestations had the highest risk of having multiple surgeries [9]. However, in our study, we have done operative intervention in 100% of patients, but the need for a second surgery did not arise in any patient.

Regarding the microbial testing of the disease, Raja et al. mentioned that many factors play a vital role in the diagnosis of Burkholderia infections: (1) sample collection should be appropriate, (2) sample transportation should be in a sterile container without any delay to the microbiology department, (3) staff who handle the sample should be trained and experienced, (4) the culture media for incubation of bacteria should be of best quality, and (5) sensitivity testing should be done with the use of specific antimicrobial disc. Strict adherence to abovementioned steps may lead to high chances of isolatingB. pseudomallei [12]. Molecular methods, for instance, polymerase chain reaction (PCR), or pulsed-field gel electrophoresis, are being employed in clinical and research laboratories; these are less sensitive than gold-standard culture results [12].

With respect to antibiotic administration and follow-up, Raja et al. stated that intravenous drug administration must be extended for deep-seated and/or complicated infections up to eight weeks, with subsequent administration of oral therapy for at least 12 weeks [12]. Shetty et al. noted that the intravenous (IV) antibiotics used were meropenem and/or ceftazidime. The representation rate was higher in patients with less than four weeks of IV antibiotics (7/18 patients), in contrast to patients with more than five weeks of antibiotics (3/32 patients) (p=0.027). All patients were given trimethoprim with sulfamethoxazole (TMP-SMX) or doxycycline, for an additional three to six months. However, no significant difference was found between the group of patients given three and six months of oral antibiotics, in view of disease representation or complications [9]. On the basis of these two studies, in our study, we have given IV antibiotics for a minimum of six weeks (average: 6.2 weeks) to all patients, followed by oral antibiotic therapy for three to six months (average: 105.6 days) on the basis of regular follow-up of clinico-hematologic parameters.

Raja et al. stated that high relapse rates inB. pseudomallei infections are already stated [12]. It is important to complete the treatment to prevent relapse of melioidosis. Furthermore, surgical drainage of musculoskeletal abscesses is the mainstay of treatment of melioidosis, in conjunction with antimicrobial therapy [17]. Treated patients require long-term follow-up due to the organism showing latency of up to 26 years in the body [12]. However, no relapses have been noted in this study, but the duration of follow-up in this study is comparatively small. Furthermore, prospectively following up with the patients will be a prerequisite to the diagnosis of relapses, if any.

## Conclusions

Burkholderia species infections should be considered as a potentially increasing causative agent of musculoskeletal infections in non-endemic areas. A high index of suspicion is required in patients with comorbidities. Isolation of clinical specimens is of utmost importance and is considered to be the gold standard. However, we discuss that a lack of diagnosis or late diagnosis of Burkholderia can lead to a more vicious musculoskeletal involvement, prompting a shift towards more aggressive surgical treatment, subsequently leading to a prolonged ICU stay. We should be more suspicious about the organism and send an extended spectrum of investigations when dealing with these types of patients. Though the number of cases and their follow-ups are not large in this study, further follow-up and research are the mainstay.
